# Ethics, Nanobiosensors and Elite Sport: The Need for a New Governance Framework

**DOI:** 10.1007/s11948-016-9855-1

**Published:** 2016-12-19

**Authors:** Robert Evans, Michael McNamee, Owen Guy

**Affiliations:** College of Engineering, Swansea University, Bay Campus, Fabian Way, Swansea, SA1 8EN UK

**Keywords:** Ethics, Sports engineering, Nanotechnology, Nanomedicine, Sport, Data privacy

## Abstract

Individual athletes, coaches and sports teams seek continuously for ways to improve performance and accomplishment in elite competition. New techniques of performance analysis are a crucial part of the drive for athletic perfection. This paper discusses the ethical importance of one aspect of the future potential of performance analysis in sport, combining the field of biomedicine, sports engineering and nanotechnology in the form of ‘Nanobiosensors’. This innovative technology has the potential to revolutionise sport, enabling real time biological data to be collected from athletes that can be electronically distributed. Enabling precise real time performance analysis is not without ethical problems. Arguments concerning (1) data ownership and privacy; (2) data confidentiality; and (3) athlete welfare are presented alongside a discussion of the use of the Precautionary Principle in making ethical evaluations. We conclude, that although the future potential use of Nanobiosensors in sports analysis offers many potential benefits, there is also a fear that it could be abused at a sporting system level. Hence, it is essential for sporting bodies to consider the development of a robust ethically informed governance framework in advance of their proliferated use.

## Introduction

Performance analysis is an essential element of elite sports success, whereby teams and coaches continuously strive for ways to improve performance and maximise opportunities for success. The ethical importance of one aspect of the future potential of performance analysis in sport, combining the field of biomedicine, sports engineering and nanotechnology in the form of the ‘Nanobiosensors’ is critically discussed here. This innovative technology has the potential to revolutionise sports, enabling real time biological data to be collected from athletes that can be electronically distributed. As with many technologies, unintended uses may detract from their overall value. More troublesome arguments concerning (1) data ownership and privacy; (2) data confidentiality; and (3) athlete welfare are presented. While not unique in biotechnology, a proper consideration of these issues must inform ethical and regulatory discussions concerning the introduction of nanotechnology in sport. Whilst the future use of Nanobiosensors in sports analysis offers many potential benefits, there is also genuine concern that it could be misused at a sporting system level. Hence, it is essential for international sporting bodies to reflect critically on the need for a robust ethical framework in advance of their proliferated use.

## The Rise of Performance Analysis in Sport

According to the English Institute of Sport, the discipline of performance analysis is a focus on developing interventions within the coaching process, allowing for performance gains and augmenting learning. It is driven by a desire at the elite level to make vital performance gains through improving tactics and techniques via real and lapsed time objective feedback (EIS N.D, para. 1). A key point is that Performance Analysis is typically third person. Research has shown that athletes and coaching teams on average only recall 30% of performance carried out by an elite sportsman, which can result in only small gains for the athlete (EIS N.D, para. 1). Performance analysis is therefore used to make up (at least part of) the 70% that was missed, ensuring that the athlete has the best feedback possible to improve their performance (EIS N.D, para. 1).

Performance Analysis is not a homogenous phenomenon, with differences arising from location, timing, and nature of information, biological and psychological for example. It can either take place after an athlete performs (on the side of a track for example), or it can take place in a laboratory, providing a more controlled environment (EIS N.D, para. 2). There are also a number of ways in which performance analysis techniques can be used in sport. For example within training using visual feedback or alternatively in a competitive environment, using profile data of the performer in order to develop a game plan ready to compete successfully (EIS N.D, para. 3). In order to provide such information, performance analysts utilise advanced video performance software systems such as ‘Dartfish’ (Dartfish N.D). These systems enable athletic advancements by enhanced analytics that encourage more critical performance feedback and reflection between coaches and athletes.

## Benefits and Disbenefits of Performance Analysis

The use of performance analysis (PA) is thought to be essential to both athletes and coaches to ensure consistent success rates in competition (SINI N.D, para. 3), especially at elite levels. The effective use of performance analysis brings benefits for coaches and athletes alike, allowing for improved tactical/decision making, greater athlete confidence and improved reflective coaching techniques (SINI N.D, para. 4). Although PA may be thought to be essential in elite sport, we must recognise both the benefits and disbenefits of their use, which are patterned in their distribution and relate both to the individual athlete and sporting system (Table 1).

**Table 1:** Benefits and disbenefits to both the athlete and sporting system

Benefits for the athlete	Benefits for the system	Disbenefits for the athlete	Disbenefits for the system
Increased performance data	Improved safety in sport	Athlete consent, confidentiality and data ownership	Unfair competition in sport—‘Technology doping’
Increased value for the athlete	Deterrence to cheating	De-skilling of an athlete	Can facilitate corruption

Having sketched out a matrix for PA generally, we turn specifically now to bioengineered PA, and more specifically Nanobiosensors.

Recent bioengineering innovations have the potential to revolutionise the PA landscape. The development of advanced feedback systems utilising sensor and image processing facilitate instant data transfer to laptops and smart devices for analysis (Haake 2013). This has allowed for far more effective feedback systems to be developed, via increased levels of data to be gained in less time (Baca and Kornfeind 2006).

We consider now how sports engineering and feedback systems have been used to benefit performance analysis in swimming. As feedback systems have become a prevalent method for training elite swimmers of the Great British Team, who have now implemented state of the art movement tracking and sensor systems. Enhanced technology has meant that multiple sensors have been integrated and developed to transmit wirelessly through water, something that has not been possible before. The principle behind this system is the ability to track a swimmer through the water wirelessly, allowing data to be generated of each of the athlete’s movements (such as body position, acceleration, and overall technique), so that feedback from coaches is immediate, detailed and objective (EPSRC 2012). The information generated is then transmitted to analysts and coaches who are able to then give feedback to make real-time adjustments during training. Prior to tracking sensors, the coaching team would have had to give feedback to swimmers through observational and video-based analysis post-training, which can sometimes lead to loss of precision and efficacy.

Despite these benefits, this form of feedback is limited, addressing only the external movements of an athlete. Such data must be augmented by, for example, blood sampling for lactates, in the middle of training for information on the relative fatigue state of the athlete. These must be analysed away from the training site. Consequently, sports engineers have looked to other fields of research such as biomedicine and engineering to help solve this problem. One solution is the biosensor, which enables biological data capture through mobile devices. Some sports systems now make use of wearable technology to record key health and performance data (Smith 2014, p. 1). Biosensors offer athletes and coaches a revolution in sport analysis; as performance data such as acceleration position, respiration rates and fatigue levels can now be collected wirelessly, allowing for continuous monitoring of an athlete is real time (Moskvitch 2012). This allows for a more comprehensive picture of the athlete’s fitness, and greater levels of insight into what their bodies’ metrics look like during training and performance. Further, the data collected though the use of biosensors can also be used to predict what may put an athlete’s health at risk, consequently supporting early diagnosis of conditions such as cardiac arrest, making sport potentially safer for the athlete (Moskvitch 2012).

In summary, we can say that biosensors offer a more bespoke approach to enhancement for athletes, whilst also enabling precise monitoring of their health profile too. Nevertheless we must ask more precisely what biosensors are and how they can or ought they to be applied in elite sport.

## Feedback Systems: Biosensors/Nanobiosensors Performance Analysis Systems

Before considering the ethical and unethical aspects of Nanobiosensors, we must first consider what a biosensor is. Currently two definitions are widely recognised. Higson defines a biosensor as ‘a chemical sensing device in which a biologically derived recognition entity is coupled to a transducer to allow the quantitative development of some complex biochemical parameter’, while Frazer defines it as ‘an analytical device incorporating a deliberate and intimate combination of specific biological element and physical element’ (Higson et al. 1994; Fraser 1994). Biosensors consist of two parts: a bioelement and a sensing element (Mohanty and Kougianos 2006). They comprise of a biorecognition element that responds to a target compound which creates a biological response, which is then converted by the transducer to a detectable signal, that is measured *inter alia* optically or acoustically (Touhami 2014). The use of biosensors can support an athlete’s pursuit of excellence, as they can be used to keep them in peak physical condition, ensuring high levels of endurance, speed and efficiency (Montgomery 2010). But to remain in this condition while training *in extremis* for extended periods is highly challenging. It is often said that good health ends where elite fitness begins. Precise, accurate and timely monitoring is therefore essential to athlete centred sport technology and sports medicine (Dijkstra et al. 2014).

An example of a biosensor used for sporting purposes is a Pulse Oximeter (Sheehan 2010). This is a handheld electronic device that is used to measure the amount of oxygen in athletes’ blood. It works by clipping the device onto an athlete’s index finger, which then radiates infrared light. The oxygenated and deoxygenated blood absorb at different levels enabling the accurate calculation oxygen is the athlete’s blood. The ability to measure oxygen in the blood is essential since optimum lung function is vital to efficient metabolism and muscle function, particularly during high intensity exercise, where the muscles are working harder for longer periods of time (Montgomery 2010). The Pulse Oximeter is used to monitor potential oxygen drops during situations such as these, and enables coaches and athletes to develop new methods to increase stamina for greater competitive gains.

Nevertheless, biosensors have limitations such as the (1) volume of biological data they are able to collect; (2) practical functionality for elite athletes in terms of size and bodily restriction; and (3) over-sensitivity that can sometimes lead to false data measurement. In consequence, sports and biomedical engineers have sought to overcome these deficiencies in the form of Nanobiosensors.

Nanobiosensors differ only in scale from biosensors in general. A Nanobiosensor may be defined as a biosensor consisting of nanomaterials and with the dimensions on the nanometer (1 nm = 10–9 m) (Nada and Shimaa 2011, p. 92). The incredibly small size of Nanobiosensors has multiple advantages, due to the large surface area to volume ratio; many of the nanomaterial atoms are located near their surface, resulting in improved transducing and signaling capabilities (Malik et al. 2013). This increases their ability to detect and provide more accurate data recording. Further, these devices can be fitted to a person without irritation, and placed either subdermally or externally on the body for extended lengths of time. One of the main reasons for this is possibly due to the production of thin films made of nanomaterials, which are able to increase the detection of molecules such as enzymes and DNA, consequently allowing for on the spot, real time monitoring, which is highly beneficial to elite athlete and their performance progression (SHL 2014).

The exciting new possibilities have opened the floor for worldwide debate in academic study, especially in relation to performance analysis (Malik et al. 2013). Although Nanobiosensors are still in the early phases of production, they are likely to transform the ways in which performance analysts use feedback systems in elite sport in real time (SHL 2014).

## An Example of Nanobiosensors Feedback Systems for Performance Analysis

Nanobiosensors offer huge potential to elite sport, but are very much in their research phase, with teams across the world developing and trialling new avenues for its use within the world of elite sport and medicine. Nanoengineers have developed a non-invasive Nanotattoo that can be placed on an athlete’s arm to monitor lactate levels of perspiration (Wenzhao et al. 2013). The monitoring of lactate is an essential indicator for assessing physical performance during training and competition in multiple sprint and speed endurance. Traditionally lactate levels have been monitored through the use of lactate sensor strips that are used with a hand held device, which is both inconvenient and intrusive during physical activity (Wenzhao et al. 2013). By contrast, the noninvasive enzymatic temporary-transfer tattoo acts as a flexible sensor made through screen printing methods, which flexes to fit the body. This ensures it is non-intrusive and resilient to cope with intensive exercises, due ‘to the presences of dispersed carbon fibres within the screen printed inks’ (Wenzhao et al. 2013). Establishing more precise measures of athletes lactate levels, a very significant performance inhibitor, enables significant improvements for athletes and coaches, as it will allow for data to be gained immediately on biological performance, enabling immediate interventions in training sessions.

Having established that the use of Nanobiosensors in elite sport is exceptionally promising, we consider now a number of ethical and legal concerns that need to be addressed fully before its widespread implementation. We turn now to a discussion the potential benefits and disbenefits raised earlier.

## Benefits for the Athlete

### Higher Value Performance Data

Previously performance analysis has been limited due to biological data not being readably available for analysis. But, with the integration of biosensors and in the future Nanobiosensors, coaches and athletes are gaining a far greater understanding of how an athlete’s body works. The future use of Nanobiosensors in the form of wearable technology will be able to wirelessly track a number of bodily functions such as rates of dehydration, recovery, lactate levels and even wound healing (SHL 2014). This will move athletes and coaches away from reliance on physical metrics, and provide a more accurate picture of an athlete’s biological function. Moreover, increasing the amount of data available for performance analysis, and allowing coaches and athletes to develop a far more bespoke approach to training, thus, creating greater potential for successful outcomes in competition (Saxon 2011). In addition, the use of Nanobiosensors increases the speed and availability of data, transmitted in real time to coaches and athletes on their smart phones or tablets, enabling immediate interventions.

### Increased Value for the Athlete

Player performance statistics have played an increasingly prevalent role in the renegotiations of contracts and salaries (Socolow 2015). As Marathe, Chief Strategy Officer of the San Francisco 49ers states, ‘good data insights can make or break a player being hired or a coach being fired’ (Brousell 2014). Nevertheless, this data is often limited to injury and basic health statistics (Socolow 2015). Consequently, it only provides an idea of what an athlete’s future performance may be, and may actually be detrimental to an athlete’s value as current performance data cannot not reliably predict an athlete’s potential.

Recent advancements in biosensors (and with confidence we may say in the near future, Nanobiosensors too) have brought significant enhancement in the levels of biological data including indicators of long-term health and future injury rates. The data can play positively into the hands of an athlete, as strong biometric data can work to increase the value of an athlete; not only to a team or organisation, but also financially to the club or franchise (Socolow 2015). For example, in the future if a football player is regularly being identified through Nanobiosensor monitoring as the most physiologically superior player in a squad, this is likely to increase their economic value. The National Basketball Players Association has already cited many examples of data from biosensor technology being used in contract negotiations (Socolow 2015). Other measures could include players that have the lowest levels of lactate production through games; the calmest under pressure with lower heart rates during critical times in the game such as penalties or vital free kicks; or often more importantly to clubs, the player with lower injury frequency. In doing so, Nanobiosensor data readings are likely to be used to further justify improved contracts, team transfers or salaries of players, particularly when comparisons can be drawn directly between the biological data of any one player to another. This could also be advantageous for clubs, as it will give coaches and managers the confidence to invest money in quality players without the fear of diminishing returns due to long-term injury, thereby reducing risk from the offset. Avoiding frequent injury of star players where possible could also contribute to increased performance outcomes on the pitch, as a side is likely to remain fitter for longer; an essential element for any team mounting a campaign in extended competitions such as UK football’s English Premier League, or the National Football League in the USA.

## Disbenefits for the Athlete

### Deskilling of an Athlete

There are concerns that the use of engineering in sports more generally may deskill athletes, creating a greater reliance on the technology (James 2011; Magdalinski 2008; Miah 2006) and generate negative connotations for both the athlete, and the sport more generally (James 2011). In a system where one values the general capacity of an athlete to self-monitor a range of capacities or performance dimensions, or to make autonomous decisions regarding how they are performing within a competition, it is clear this deskilling is undesirable. It has the ability to alter a way a sport is played or carried out, as it can change the training required to learn a new skill (Miah 2006). There are numerous examples of these types of technologies which blur this issue, such as depth finders in fishing to make it easier to locate fish, as well as U-groove golf clubs, which allowed a player greater accuracy when taking a shot (Hardman 2001; Miah 2006). Yet both were banned from their respective sports, as it was felt that they undermined key skills constitutive of elite performance, leading to a devaluation of the challenge that was thought central to the activities (Miah 2006).

A similar concern arises over the use of Nanobiosensors in the future to maintain an athletes biological function. Self-monitoring is an important aspect of being an elite athlete, evidence can be shown in the ability to control one’s diet; know one’s limits, or find ways to adapt training approaches when extant training or performance strategies are sub-optimal. It can be argued that the underlying competencies for these abilities are part of what separates the elite athlete from the merely good performer. Nevertheless, if sensors such as Nanobiosensors provide the level and speed of precise data, which coaches interpret and base interventions upon, one may ask in addition whether the traditional skill set of the coach and athlete will be lost, or perhaps transformed? For example, when athletes are training, they require a skill level to work out when would be an ideal time to stop before they risk injuring themselves. The athlete wearing a Nanobiosensor, appears to render this competence redundant. The Nanobiosensor would inform them when they need to stop, instead of an athlete trusting in their own knowledge of their body. A further example could be diet, an area in which many athletes are skilled in, knowing what exactly they need to eat and when, in order to ensure their body continues to perform the way they want it to for successful outcomes. Yet if an athlete is wearing a Nanobiosensor that can inform them of the nutrients they require at the point of optimal need, it will remove the judgmental capacity an athlete requires when implementing and developing an effective dietary plan. Hence, the use of Nanobiosensors could potentially devalue sport, through reducing the skill required of an athlete to take part at elite levels, much like the U-groove golf clubs and depth finders mentioned earlier. Finally, some sports value the individual autonomy of their athletes more than others. Thus in tennis, coaching from the sidelines is forbidden by the rules. Once the player steps on the court it is him or her alone against their opponent. This is in stark contrast to, say, American Football where technology intervenes more readily and more frequently, diminishing player autonomy and enhancing system control. Whether enhancing system control over athletes is a good or bad thing for sports is a moot point (Loland 2002) and ought to be considered on a case-by-case basis.

As a consequence, the use of technological innovation has resulted in performance comparisons in many sports becoming less meaningful, as some argue that technology has enabled athletes to surpass records set by previous masters with relative ease, without having to develop the same level of skill. Some feel that this is not only disrespectful to these previous masters of a sport, but against continuity of sporting traditions (James 2011). This is a point similarly discussed by Carr (2008) who argues that the role of sports engineering is to support the development on an athlete, *not* to remove the need to develop key skills to be successful at a sport which people expect from an elite athlete, ‘as that would be inconsistent with the point or purpose of play’. Therefore, it is essential that Nanobiosensors be used to support the athlete, not remove the level of skill required to be an elite athlete.

### Consent, Confidentiality/Privacy and Data Ownership

The current use of biosensors, and the future use of Nanobiosensors for performance analysis, raises serious concerns surrounding the gathering of biological data from an athlete. As despite the benefits Nanobiosensors may bring to an athletes performance in the future, the benefits of this technology must be carefully balanced against key issues such as privacy, security, consent and data ownership (Meingast et al. 2006). This kind of data could be highly sensitive, and may be considered more private than statistical data currently being collated in sport.^1^ This could create highly complex issues for organising bodies, as the path to clear and controlled regulation of Nanobiosensors in sport could prove highly complex.

Consider the issue of consent in regards to Nanobiosensors, as due to the complex nature of the technology and the knowledge gaps we have surrounding how nanoparticles alter human physiology. There is a concern over whether we possess sufficient understanding of the technology to allow for consent to be properly informed (Brewer and Gurel 2009). If we refer to the field of medicine, before a doctor can carry out a procedure or treatment, they must seek patients’ consent, to reasonably inform them of the details, risk and benefits associated with a given treatment before it can go ahead (Kegley 2004; NHS Choices N.D). Where a reasonably complete set of details regarding the procedure cannot be provided, then the patient cannot be considered to have given *informed* consent (Kegley 2004). A similar train of thought can also be applied to use of Nanobiosensors in elite sport, though in vivo effects are not well understood. However, early studies have indicated that they may pose a risk to human health due to their unique size, allowing them to enter the body via channels such as the respiratory system, skin or mouth (NIOSH 2009). Once in the body, they then have the potential to cross major bodily systems such as the blood brain barrier, posing serious health risks. Early evidence has indicted that nanomaterials have the potential to be toxic to human tissue and cell cultures (Madhwani 2013). As a consequence, this could mean that the health of athletes and players who wear Nanobiosensors may be at risk. Further studies are required to discover the true extent of the potential risks associated with exposure to nanoparticles, which means until this knowledge gap is truly bridged, teams and organisations will not be able to provide a reasonable catalogue of the risks and benefits of using Nanobiosensors, resulting in players and athletes both ethically and legally being unable to provide their informed consent.

In addition, due to the potentially sensitive nature of information gained by the use of Nanobiosensors, one might presume that a player should be part of a consensual agreement for its wider use, with third parties such as agents or physicians, as the data is collected and used in a manner that is not anonymised (Meingast et al. 2006). Its use may be either beneficial or prejudicial to their interests. As ethical consideration of the use of data that may be prejudicial to the interests of individual athletes must be directed to the issues of ownership and storage. Who owns such data and consequent information flows? Do they belong to the athlete or the club or organisation they represent? For example, according to the European law at present, ownership is dependent on who physically collects the information in the first instance. In our hypothetical case this is likely to be the clubs or potential organisations athletes represent, who would own the raw data and any analysis that takes place at a later date (Socolow 2015). This also raises further questions of how this data would be stored once collected (Meingast et al. 2006) and in what form. Should the raw data be collected and then stored locally or centrally? Which of these options offers better security in relation to the privacy needs to an athlete, to ensure that their information is not lost of stolen. Further, who would be able to access this database and thus access, alter or remove the athlete’s private records? Would there be a system put in place for this, such as the one used in medicine, where there are two categories for viewing records, those with read/write privileges (doctors and physiotherapists who may read and edit files) and those with read-only privilege (insurance providers for example) (Meingast et al. 2006). This is a key point that must be addressed before Nanobiosensors are fully integrated into sport.

Thus, there are serious concerns over the use of Nanobiosensors in relation to privacy and confidentiality. As stated earlier, the use of WI-FI Nanobiosensor would alter the way in which athletes traditionally collect their biological data records, something not fully possible with current biosensors (although some are trialling this now), enabling instant data transfer. However, the potential use of WI-FI to transfer athlete biological data does raise concerns over security, as it exposes this data to greater levels of attack, through data hacking for example, leading to further concerns over privacy. For example, athletes’ physiological vital signals are sensitive, and could identify that they may have an embarrassing disease or a career ending condition, so any leaking of this information could be deleterious to their interests (Kumar and Lee 2012). Paper based records enabled easier restrictions to be placed on access to data. Once the information is made available electronically, it may be easier for hackers to gain access and potentially increase the number of people who are able to view this sensitive information, which could be used against an athlete and potentially limit their career. In the context of elites sport, the recent Angry Bears hacking of the World Anti doping Agency’s ADAMS database shows how athletes’ medical records were posted online within minutes of being accessed by Russian hackers aggrieved at their country’s expulsion form the Rio 2016 International Paralympic Games. Furthermore, wireless sensor networks also bring considerable challenges to ensure the integrity of the data collected, as eavesdropping and skimming are a greater possibility when data is wirelessly transmitted (Meingast et al. 2006). This could make it a lot easier for opposing teams or athletes to be able to access another athlete’s biological data in the hope of gaining an advantage over them. Therefore, it is vital that security and encryption services are well thought out when integrating this technology into elite sport. As if its not, it may generate social unrest amongst athletes, who may feel that their private and sometimes sensitive biological data could be at risk through hacking or misuse by private organisations, leading to potentially negative impacts on their careers (Al Ameen et al. 2010).

A further point regarding data security arises in the contexts of obligations to safeguard a player against third parties such as the broadcast media, or even video game creators, acquiring such information, or selling this on for profitable gain. Moreover, would clubs, teams and organisations be obliged to share the statistical analysis of data collected with the athlete or coaching team in question to enhance their own performance, or would there again be a cost implication involved? Each of these scenarios create a significant privacy and confidentiality issue, which athletes must be made aware of in relation to performance and training but also key encounters such as contract negotiations. It is also important not to blur the line with athletes, and simply view them as guinea pigs for sports’ biotechnological development (Comporesi and McNamee 2014).

Finally, the continual wearing of Nanobiosensors raises a concern about the blurring of the private/public distinction. This is highly relevant to occupational scenarios. When is an employee required to conform to their employer’s demands? Of course just about any clause for performance of duties may be written into a contract and consensually signed. Yet if monitoring 24/7 is permitted, one wonders if the notion of a private life is rendered redundant. For example, Brian Buckle (a defensive lineman in the Canadian Football league) has referred to himself and his teammates as guinea pigs for new technologies, whose right to a private and family life (e.g. Article 8 of the European Charter for Human Rights) is compromised (Lindzon 2015). In summary, there are important issues to be evaluated before the integration of Nanobiosensors to elite sport.

## Benefits for the Sporting System

### Improved Safety in Sport

The development of sports engineering has been a significant driving force behind the development of safety in sport. Bjerkile (1993) considers the example of fencing, where sports engineering has made safety standards for clothes much more stringent. For example in fencing, jackets are now made of Kevlar, and masks constructed of stainless steel with a stronger mesh that can withstand puncture tests. This has also proved beneficial to the public who decide to take up the sport, as it is viewed as a safer way of practicing, reducing the likelihood of a person receiving an injury.

The growth of Nanobiosensors in the future may promote greater safety in sport (Saxon 2011). The use of biosensors such as these can bring about a safer playing field, through helping to avoid injury, as their increased sensitivity can be used to check an athlete’s vital health data, ensuring athletes conform to agreed risk standards. To demonstrate how biosensing technology such as Nanobiosensors could be used to promote safety in sport, Saxon (2011) carried out study the changing heart rates of NFL players throughout a real game situation using heart rate patches gathering data from a tough scrimmage game that involved continuous changes in acceleration and significant physical contact. This form of monitoring was a breakthrough in the field, of which the purpose of the study was to gain baseline heart rate data for each player to establish what would be deemed ‘normal heart rate’, and ‘abnormal heart rate’ for a player in their position. Establishing such data proves beneficial for player welfare, allowing athletes to be monitored throughout a game in real time, also enabling the coaching team to react in a prompt and effective manner when required. It may also prove highly beneficial in gruelling pre-season preparations.

Therefore, the potential benefits that this new technology can bring to the sport are significant, not only through enhanced performance analysis, but also by providing vital statistics that could prevent long-term injury and increased safety for athlete welfare.

### Deterrence to Cheating

Ever since competitive sport began at the time of the ancient Greeks, professional cheating has always been prevalent and has ever since become a feature the sporting landscape (Møller et al. 2015). There are many ways in which athletes and coaches can cheat, however in recent years doping has certainly been the most widely publicized.

At present, the main method of testing for doping substances is mass spectrometry, a method whereby electrons are fired at urine samples to ionise them, as each substance contains what is known as a fingerprint, such as a specific weight, which scientists can easily detect (BBC 2015). Like all analytical techniques, this process has its flaws, as some new drugs have been specifically designed to evade this process, and are two small for detection through mass spectrometry (BBC 2015). Moreover, the practice of micro doping has made detection exceptionally difficult. As a result, it has become far more difficult to detect, and therefore punish those athletes who are using prohibited substances and methods who illegitimately enhance performance.

The increased development and use of biosensors and Nanobiosensors may bring greater balance to the cheat: regulator balance, through increased sensitivity. Deviation from normal functioning for a particular athlete has been made more plausible by the Athletes Biological Passport, which takes measures over time (e.g. blood variables) where anti doping laboratories can look for peaks in biological markers and correlate them with athletes whereabouts data (MacGregor et al. 2013), thus inferring when athletes have gone on a “doping holiday”, for example, in parts of the globe where they are difficult to track down by doping control officers.

Nanobiosensors could be used to further enhance this capability due to their potential for increased sensitivity and greater live-time monitoring. This could be highly beneficial to raising trust in sport, especially in sports such as athletics and cycling, where doping scandals have damaged the reputation and commercial value of sport. The use of Nanobiosensors could be viewed by spectators as creating a fairer sport and a level playing for the athletes, through further reducing the chances for athletes and coaches to dope.

## Disbenefits for the Sporting System

### Unfair Competition in Sport: ‘Technology Doping’?

The idea of technological doping is a relatively new one, referring to the advantages that might be brought to an athlete through using technology (PDD 2014). This has consequently raised the question of ‘when does the competitive edge stop and unfair advantage begin?’ (PDD 2014). After all, sport is a competitive business and if, for example, the British cycling team can build better bikes than other countries, it can be argued, should this not be allowed insofar as their scientists and engineers are working within extant regulations and parameters? This raises questions of relative fairness in contrast to equal opportunities to compete. This has been referred to as the ‘fair opportunity principle’ (Loland and Hoppeler 2012), which may apply, where a team although working within permissible parameters, gains so significant an advantage that it renders the competition unfair. Most recently, the ruling body for European Football, has attempted to diminish the effect that wealthy owners have had on winning teams by adding a new Financial Fair Play policy (UEFA 2012), which demands that clubs cannot continue to lose money while being underwritten by unlimited financial means (Preuss et al. 2014) over those clubs who stay within their commercial budget. In the USA, a draft system attempts to ensure some relative fairness in playing resources is provided as an alternative model.

The issue of fairness and use of technology in sport could also be raised in relation to Nanobiosensors. We have noted above how this form of technology can offer athletes access to higher levels of biological data for performance analysis; something that could not be gained through traditional methods, consequently resulting in them being able to develop a much more bespoke approach to their training and potentially gain new levels of success. We may reasonably assume that not all athletes will have access to these sensors due to high patent costs, resulting in a potential unfair advantage to those who have them. For example, nanobiosensor feedback during a marathon, or endurance cycling race (such as the Tour de France) could use Nanobiofeedback to tweak tactices during the race. The coaching team may notice for example that the athlete is running low on glucose, and so could advise them to take a glucose providing drink as soon as possible to maintain consistent energy levels, which could be the difference between winning or losing. Therefore, taking away the traditional skill a marathon runner requires to judge when they need to re-energize or not, and potentially providing an unfair competitive advantage.

There is also a concern that the increased integration technologies such as Nanobiosensors into sport using advancements may not be a test of an athlete’s abilities, but rather the strength of the technology systems (Loland 2002; PDD 2014). This may lead to a technological “arms race”, where those teams with the best resources will always have an advantage over others, a fear that many are currently having with Formula 1 motor racing, whereby the differences between the drivers are actually minimal, but every year the team with the most advanced technology wins. Hence, it is important to monitor the application of technology in sport, to ensure that competitions remain fair for all athletes involved.

### Corruption

If doping is the biggest threat to the integrity of sport, then match fixing or event manipulation must be a close second (McNamee 2013). The use of Nanobiosensors for performance management brings many potential benefits, as noted earlier, yet their use within elite sport also has the potential to increase corruption in sport. Enabling real-time transfer of an athlete or team’s biological data gained through future WI-FI enabled Nanobiosensors, might expose teams and athletes to data hacking, with key information being stolen or leaked deliberately to the opposition. This would give a new application to the saying ‘stealing another coaches playbook’ (Socolow 2015). For example, in rugby, a team’s biological data could be hacked and shared with the opposition, allowing them to adapt their tactics in the hope of improving success rates. They may for instance discover that a key player runs low on glucose levels at certain points in the game, suffers from fatigue, or has a recurrent injury—and can therefore adapt their team play accordingly to target such player weaknesses, in the hope of swaying the result in their favour. Increasing the opportunity to improperly manipulate game outcomes or “match-fix” (manipulate the outcome of the event) through stealing biological data is a domain that lacks a specific and universally adopted regulatory body or framework. It is questionable whether sports’ existing regulatory frameworks for match fixing (event manipulation) could respond to this phenomenon or whether criminal prosecutions in relation to data law would have to be pursued.

In addition, there are a number of labor concerns over the use of Nanobiosensor, raising the potential for corruption to take place at a system level. As referred to earlier, athletes could potentially use the statistical data from the Nanobiosensor performance analysis for positive reasons, such as strengthening their contractual situation with excellent biological data, demonstrating a positive investment for a club or organization, though equally possible a negative effect from athlete’s perspective. For example, if a football player’s data showed poor biological trends, this could work against them when signing for a new club or renegotiating new contracts. If a trend emerges that a specific player becomes fatigued during most games at around the 50-minute mark due to lactate build up, then this could actually reduce their value, as clubs could argue that they can get another player who has better biological endurance data, and in doing such, forcing a player into a diminished economic contract or cessation of employment. Furthermore, if a player is looking to move clubs, this information could be used against them as, no matter how well they perform on the pitch, their predisposition for early onset fatigue could deter a club from employing them. This information could simply exacerbate periods of poor performance, which may not last indefinitely, but could affect their economic value. It could also add a lot of stress to an individual, as if they know that their biological performance data could be used against them in this way, it could also result in detrimental performance outcomes in matches, and more broadly an athlete’s career. Moreover, clubs could use this information to blackmail key players into staying at a club, as they could threaten to release potential damaging information such as future injury to other clubs, which could make a player less attractive. Nevertheless despite the potential positives Nanobiosensors could bring to an athlete financially, it could also impact on them negatively, and have a detrimental effect on their financial situation as well as future employment.

In preparation for the adoption of Nanobiosensors into elite sport, it is vital that regulatory bodies and sporting organisations confront the potential disbenefits raised by this technology. There are a number of ways in which this can be done, as some of the issues raised by this technology are no different to those presented by previous technologies, thereby allowing the use of current regulatory measures to be applied. However, Nanobiosensors also present a number of issues that previous technologies have not. Therefore regulatory frameworks will be required to effectively manage these technologies in sport. In this section we propose a number of ways in which we can move forward with Nanobiosensors in sport, taking into consideration both current and future solutions.

## Existing and Potential Solutions to the Ethical Problems of Nanobiosensors in Sport: Use of the Precautionary Principle?

In regard to issues concerning consent, regulatory bodies could address this, for example, in a similar way to the implementation of the UK’s NHS Act of 2006, Data Protection Act, and Human Rights Act in medicine (NHS England 2012). An athlete could sign legal documentation to ensure that their biological data is protected from misuse, stored correctly, and used only when authorised to ensure it is not used to their detriment. This could be built into new contractual agreements in preparation of its use, to ensure that all athletes’ rights are protected. In addition, a role-based access control could be used to protect any data stored, as it decreases the complexity and major cost of securing a potential large data storage network (Meingast et al. 2006). Again, these are often used in healthcare services to protect patient data and ensure confidentiality, and the avoidance of eavesdropping or data skimming. Thus, for example, an encryption service could be based on extant technology such as the TinySec, which has been created with sensor networks in mind, and is able to both encrypt and authenticate data for additional protection (Meingast et al. 2006). This sensor protection system is already being applied to the medical world. Further, a team of independent adjudicators could be used to apply, monitor and ensure the correct implementation of Nanobiosensors to deter it’s misuse, and corruption of the sports industry. Therefore, there are a number of solutions that can be put in place now to potentially deal with the future integration of Nanobiosensors into elite sport. Nevertheless, these fixes do not deal with all the potential issues that this technology raises, therefore, future solutions should be considered to ensure full potential concerns are covered and dealt with ethically.

One of the main future developments for the integration of Nanobiosensors into elite sport is the development of a regulatory framework and policy for its use. This is necessary, as a flexible set of regulations will further protect both the athlete and the system to foul play. Despite the existence of regulatory frameworks presently available to protect an athletes data and thereby confidentiality, concerns over privacy and consent remain. Therefore, it is important that sports regulators work together to develop a governance framework that can be put in place in preparation for this technology. This will ensure a ‘pre-mortem’ has been done to prepare for potential future complications, instead of applying the technology then reacting when complications occur, which can often be significantly more difficult to deal with at a later stage.

A regulatory framework that sets limits on how these sensors are used in athletes’ private lives will be necessary. We may reasonably foresee the 24/7 cycle of data collection to exploit Nanobiosensor potential. This would be heightened during peak competition times, but gathering important non-performance data even at rest. Furthermore, a well-planned security network needs to be generated and deployed to protect the wireless network that the Nanobiosensors will be working off from potential hacking, and protect an athletes biological data, ensuring privacy and confidentiality (Kumar and Lee 2012). This will ensure athletes have greater levels of trust in using Nanobiosensors, and therefore be more willing to buy into their use. Consequently, these are key aspects that must be explored and developed before its integration into elite sport takes place, ensuring all future concerns have been considered fully.

However, until sporting bodies mobilise governance resources there will be a gap in regulatory frameworks. The early development and adoption of Nanobiosensors presents a critical period in terms of risks to athlete welfare and the justice of sporting systems. Further, it could also result in the ethics of this technology falling behind its research and development, which could mean that when a regulatory framework is finally put in place, it may be unfit for purpose. With this in mind, it is essential that precautionary measures are taken during any interim stages to promote the safe and ethical use of Nanobiosensors, both for athlete welfare and the elite sport system as a whole. This will also help to raise the profile of ethics within technological development in sports, so that it is seen as an essential element of sports governance.

There are many ways in which this interim period could be bridged to ensure the safe protection of both the individual athlete and the sporting system; one of which being the application of the Precautionary Principle (CEE 2000). This states that if there is likely to be serious and irreversible damage to either humanity or the environment through using a technology, then lack of full scientific evidence of risk should *not* be a justifiable reason to develop or utilise it (CEE 2000, p. 10). The use of this principle would also fall into line with other countries’ and organisations’ approaches to governing nanotechnologies (such as the European Commission’s Voluntary Codes of Good Conduct for Nanotechnologies), whilst specifically developed frameworks are being discussed and developed in the future (O’Mathúna 2010). One of the main reasons for the application of the Precautionary Principle in this circumstance, is that it will provide a level of protection to athletes and sport from the unknowns which are related to the potential use of Nanobiosensors in elite sport. This is especially needed when we are discussing the potential health risks to athletes who wear them. The infancy of nanotechnology coupled with a lack of human clinical trials means that there is very little scientific knowledge of how Nanobiosensors may interact with(in) the human body over time. Hence, effective precaution must be taken to ensure that an athlete’s safety is put at upmost importance. Further, the Precautionary Principle may also protect athletes from misuse of their biological data helping to limit any potential corruption within the sporting system as a whole, or risk to their professional and personal lives.

Finally, it may be argued that the application of the Precautionary Principle to the use of Nanobiosensors within sport could result in a blanket ban as soon as it presents *any* risk to an athlete or player, as its purpose is to prevent risk and potential harm to human life. Such an undifferentiated response, is likely to be unwarranted, given the reasonably foreseeable benefits Nanobiosensors offer, and would unjustifiably hold back scientific development. Thus, each new dis/benefit should be considered individually and thoroughly on a case-by-case basis (O’Mathúna 2010), so that data can be gathered to help bridge the knowledge gaps we have surrounding their use in sport, and to determine whether the application of the Precautionary Principle is necessary. This would stop the Precautionary Principle from being used as a blanket defense, and allow it to become tailored to each case, and support the application of the right level of precaution in each case.

## Conclusion

Performance analysis is an essential training and competitive tool for any elite athlete, ensuring that they continue to attain essential marginal gains through reflective feedback practices. It is also a continually evolving practice, incorporating fields such as sports engineering and biomedical research to ensure that athletes receive cutting edge technology to allow for greater levels of analysis and feedback. This can clearly been shown through the integration of biosensors into sporting performance analysis, allowing biological data to be gained for new levels of feedback on an athletes performance in both training and competition. This integration has already started to provide significant benefits to athletes in the sporting world, and the future developments of Nanobiosensors is only likely to further enhance this; allowing athletes to reach new levels of success. Nevertheless, before Nanobiosensors become an everyday tool of sporting performance analysis and enhancement, considerations of their disbenefits—in terms of data access, ownership, confidentiality, privacy, and also athlete welfare, must be taken into account by sporting regulatory bodies to consider the impact they may have not only on the athlete, but also the sporting system. This will allow for a more considered approach, putting the value of sport and the athlete at the forefront, instead of blindly supporting the drive to introduce new biotechnologies (McNamee 2007). Hence, it is essential for sporting bodies and regulators, to develop measures to ethically integrate this technology into sport and consider the development of a robust governance framework in advance of its proliferated use within the sporting world.

## Footnotes

1 Not all authors agree on the conception of privacy this entails. For a contrast see McFee (2010).
